# Altered functional connectivity of brain regions based on a meta‐analysis in patients with T2DM: A resting‐state fMRI study

**DOI:** 10.1002/brb3.1725

**Published:** 2020-06-18

**Authors:** Dongsheng Zhang, Jie Gao, Xuejiao Yan, Min Tang, Xia Zhe, Miao Cheng, Weibo Chen, Xiaoling Zhang

**Affiliations:** ^1^ Department of MRI Shaanxi Provincial People’s Hospital Xi’an China; ^2^ Philips Healthcare Shanghai China

**Keywords:** functional connectivity, magnetic resonance imaging, resting state, type 2 diabetes mellitus

## Abstract

**Objective:**

To explore the neural mechanisms of brain impairment in type 2 diabetes mellitus (T2DM), abnormal changes to the functional connections between brain regions in the resting state were investigated based on a meta‐analysis.

**Methods:**

Resting‐state functional magnetic resonance imaging (fMRI) and neuropsychological assessment were performed on 38 patients with T2DM and 33 healthy controls (HCs). Functional connectivity between regions based on a meta‐analysis and other voxels in the brain was calculated and compared between the two groups using a two‐sample *t* test. A correlation analysis was conducted between clinical/cognitive variables and functional connection values from the regions with significant differences in the above comparison.

**Results:**

Patients in the T2DM group showed a significantly decreased functional connection between the right posterior cerebellum and the right middle/inferior occipital gyrus, left middle temporal gyrus, left superior frontal gyrus, left middle frontal gyrus, left insula, left precuneus, and right paracentral lobule/left precuneus when compared with HC group. The functional connection values between the right insula and left medial frontal gyrus, left supplementary motor area, and between the left lingual gyrus and right middle/inferior occipital gyrus in patients with T2DM were significantly decreased. Moreover, the functional connection values between the right posterior cerebellum and left middle frontal gyrus, and between the right posterior cerebellum and left precuneus were negatively correlated with HbA1c in the T2DM group (*r* = −.356, *p* = .03; *r* = −.334, *p* = .043).

**Conclusions:**

Our study showed a wide range of cerebellar–cerebral circuit abnormalities in patients with T2DM, which provides a new direction to investigate the neuropathological mechanisms of T2DM from the perspective of the cerebellum.

## Graphical abstract

The posterior cerebellum is involved in a variety of brain function impairments in T2DM patients.It is suggested that the cerebellar–cerebral circuit may be involved in the neuropathological basis of brain dysfunction in patients with T2DM, which provides new insight into the neural mechanisms of brain dysfunction in patients with T2DM from the perspective of the cerebellum.

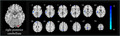

## 1 INTRODUCTION

Type 2 diabetes mellitus (T2DM) is one of the most common chronic diseases that can lead to cognitive dysfunction in attention, executive function, visual information processing, etc. (Macpherson et al., 2017), potentially increasing the risk of dementia (Biessels, Deary, & Ryan, 2008). So far, the neural mechanisms that underlie brain impairments in patients with T2DM remain unclear.

Abnormal neuronal activity is widely recognized as the neural basis of cognitive impairment (De Felice & Lourenco, 2015; Iyalomhe et al., 2017;). Resting‐state functional magnetic resonance imaging (fMRI) is a tool that can be used to explore the spontaneous activity of neurons and has been widely used to examine the pathogenesis of various neuropsychiatric disorders (Avissar et al., 2017; Cheke et al., 2017; Du, Fryer, et al., 2018). During past years, several studies have used regional homogeneity and amplitude of low‐frequency fluctuation to explore spontaneous brain changes in patients with T2DM (Chen, Liu, & Ma, 2014; Cui et al., 2015; Liu et al., 2016; Peng et al., 2016; Wang et al., 2014; Xia et al., 2013; Zhou et al., 2014), but the results were inconsistent. Zhang et al. (2018) performed an activation‐likelihood estimation (ALE) meta‐analysis and found that the left lingual gyrus, right posterior cerebellum, left postcentral gyrus, and right insula had abnormal activity in patients with T2DM in a robust way during the resting state. These brain regions are not only involved in cognitive function (Guell, Gabrieli, & Schmahmann, 2018; Stoodley & Schmahmann, 2009), but are also closely related to the clinical manifestation of T2DM, such as abnormal feeding behavior (Woolley et al., 2007). Therefore, a comprehensive investigation into the effect of regional brain dysfunction on whole‐brain function could provide more useful information to understand the neural mechanisms of brain impairment in patients with T2DM.

Resting‐state functional connectivity, which is based on synchronous and low‐frequency (0.01–0.08 Hz) fluctuations of blood oxygen level‐dependent fMRI (Biswal et al., 1995), is commonly used to evaluate interregional cooperation between different brain regions. A number of previous studies have used the posterior cingulate cortex (Musen et al., 2012), hippocampus (Sun et al., 2018), thalamus (Du, Fryer, et al., 2018), and amygdala (Xia et al., 2018) as regions of interest (ROI) to reveal abnormal functional connectivity in the brains of patients with T2DM from different perspectives. However, the choice of ROI in these studies was hypothesis‐driven. Although this approach has the advantage of providing direct information regarding the network of regions that are most strongly correlated with the seed region (Cole, Smith, & Beckmann, 2010), hypothesis‐driven ROI depends on subjective experience and prior knowledge less objectively than data‐driven ROI (Du, Fryer, et al., 2018). To the best of our knowledge, no studies in patients with T2DM have used data‐driven ROI to explore their functional connectivity with the whole brain.

Hence, in the present study, we used data‐driven ROI based on the results of a meta‐analysis, as these could more comprehensively reflect abnormal functional connectivity in brain regions in patients with T2DM. We speculated that, compared with healthy controls (HCs), brain regions in patients with T2DM have disrupted functional connectivity with the whole brain, which may reveal the neural mechanism of diabetic brain damage.

## 2 MATERIALS AND METHODS

### 2.1 Study population

From February 2018 to January 2019, we recruited 40 patients with T2DM from the Department of Endocrinology at Shaanxi Provincial People's Hospital and 35 HCs from the community. Patients were diagnosed according to the criteria of the American Diabetes Association in 2014. All subjects were aged between 40 and 70 years and were right‐handed. Exclusion criteria in both groups included a self‐reported history of known brain injury, epilepsy, stroke, alcohol and other substance dependence, Parkinson's disease, major depression, or other disorders that could affect cognitive function, major medical illnesses (e.g., cancer), and MRI contraindications. Patients with hypoglycemia (blood glucose < 3.9 mmol/L) or hyperglycemia (blood glucose > 33.3 mmol/L) during the hospital stay were excluded from the study and the examination was not rescheduled. Two patients and two HCs were subsequently excluded as the limits for head motion were exceeded during data preprocessing.

All patients took medicines or insulin injections on time on the day of the scan and arrived at the department for MRI between 06:30 and 7:00 p.m. after dinner. First, structured clinical interviews and a series of psychological tests were carried out for approximately 30 min. Patients underwent MRI within 2 hr of dinner and required a postprandial blood glucose concentration of <33.3 mmol/L. The postprandial blood glucose concentration of patients in this study ranged from 7.9 mmol/L to 21.8 mmol/L. Only one patient was scheduled for MRI each day to ensure that each patient's MRI scan was completed between 7:30 and 8:30 p.m. Eight of the 38 patients had no complications, and the remaining 30 patients had one or more complications. The complications of patients with T2DM and the therapeutic agents used to treat them are shown in Table 1. The study was approved by the Ethics Committee of Shaanxi Provincial People's Hospital. All subjects were informed of the study protocol and informed consent was obtained from each patient before participation in the study.

**TABLE 1:** Demographic, clinical, and cognitive data in the T2DM and HC groups

Items	T2DM group (*n* = 38)	HC group (*n* = 33)	*p*
Age (years)	55.71 ± 6.32	54.01 ± 4.99	.231
Gender (M/F)	30/8	26/7	.987
Education (years)	13.29 ± 2.50	15.18 ± 1.88	.001*
Disease duration (years)	8.13 ± 5.88	–	–
BMI (kg/m^2^)	24.59 ± 2.81	24.61 ± 3.10	.970
Systolic BP (mmHg)	126.21 ± 12.82	123.24 ± 8.66	.264
Diastolic BP (mmHg)	79.63 ± 9.84	82.18 ± 6.10	.202
HbA1c (%)	8.10 ± 1.86	5.67 ± 0.54	<.001*
FBG (mmol/L)	9.16 ± 2.90	5.37 ± 0.87	<.001*
TG (mmol/L)	2.18 ± 1.28	1.74 ± 1.17	.495
TC (mmol/L)	4.67 ± 1.53	4.93 ± 0.92	.396
MMSE	28.00 ± 2.01	28.46 ± 1.60	.301
MoCA	26.50 ± 2.67	27.02 ± 1.48	.311
TMT‐A	74.50 ± 27.04	71.58 ± 26.51	.866
CDT	17.88 ± 6.94	19.36 ± 5.70	.333
T2DM complications			
Retinopathy	8	–	–
Peripheral neuropathy	21	–	–
Nephropathy	22	–	–
T2DM therapeutic agents			
Dietary restriction	12	–	–
Oral medication	18	–	–
Insulin	2	–	–
Insulin + oral medication	6	–	–

Abbreviations: BMI, body mass index; CDT, Clock Drawing Test; FBG, fasting blood glucose; HbA1c, glycated hemoglobin; MMSE, Mini‐Mental State Examination; MoCA, Montreal Cognitive Assessment; TC, total cholesterol; TG, triglycerides; TMT‐A, trail making test A.

* *p *< .05.

### 2.2 Data collection

#### 2.2.1 Biochemical characteristics

Medical history and clinical data from patients with T2DM were obtained from medical records and questionnaires. Clinical data from HCs were also collected from the outpatient medical examination center, which included weight, height, blood pressure, and body mass index (BMI). Blood pressure was measured while sitting at three different time points during the day and then averaged. After an overnight fast of at least 8 hr, blood samples were obtained to measure the levels of fasting blood glucose (FBG), triglycerides (TG), total cholesterol (TC), and glycated hemoglobin (HbA1c).

#### 2.2.2 Neuropsychological tests

Neuropsychological tests were used to evaluate participants’ general mental statuses and cognitive domains. The Mini‐Mental State Examination (MMSE) and Montreal Cognitive Assessment (MoCA) were used to assess general cognitive function. The information processing speed was tested using trail making test A (TMT‐A). Visual space, visual memory, and executive function were evaluated using the Clock Drawing Test (CDT). Neuropsychological tests were carried out by a psychiatrist.

#### 2.2.3 MRI measurements

MRI scans were performed using a 3.0 Tesla MRI scanner (Philips Ingenia) using a 16‐channel phased‐array head coil. All subjects were instructed to keep their eyes closed and to stay awake during scanning. Foam pads and headphones were used to control head motion and decrease scanner noise as much as possible. Conventional T2‐weighted images and fluid‐attenuated inversion recovery scans were acquired to exclude visible brain lesions. Sagittal three‐dimensional T1‐weighted images were acquired with the following parameters: repetition time (TR) = 7.5 ms, echo time (TE) = 3.5 ms, flip angle (FA) = 8°, field of view (FOV) = 250 × 250 mm^2^, matrix = 256 × 256, slice thickness = 1 mm, no gap, and 328 sagittal slices. Resting‐state functional blood oxygen level‐dependent images were obtained using a gradient‐echo planar sequence with the following parameters: TR = 2,000 ms, TE = 30 ms, slices = 34, thickness = 4 mm, gap = 0 mm, FOV = 230 × 230 mm^2^, matrix = 128 × 128, FA = 90°, and 200 volumes.

Functional data analyses were conducted using DPABI 2.3 programs based on statistical parametric mapping 12 (SPM12, http://www.fil.ion.ucl.ac.uk/spm). After discarding the first 10 time points, the slice timing and realignment for head motion correction were performed. Any subjects with a head motion of >1.5 mm or translation of >1.5° rotation in any direction were excluded. Normalization was then performed based on the resulting images using unified segmentation of anatomical images (resampling voxel size = 3 × 3 × 3 mm^3^). Multiple regression models were employed to remove the effect of covariance of no interests, which included 24 motion parameters, cerebrospinal fluid signals, and white matter signals. The obtained images were smoothened with an isotropic Gaussian smooth kernel with a full width at half maximum of 6 mm, followed by detrending and filtering (0.01–0.08 Hz) in order.

The ROI was obtained from the previous ALE meta‐analysis (Zhang et al., 2018). ALE meta‐analysis data processing was completed in the MNI standard space coordinate system, and the results were corrected using the false discovery rate (*p* < .05, cluster size of >200 mm^3^). We presented the corrected ALE image results in the MNI standard space using DPABI software and saved the four clusters in the results map as MASK. The four ROI were the left lingual gyrus (−4, −74, −2, cluster size = 800 mm^3^), right cerebellar posterior lobe (28, −184, −14, cluster size = 488 mm^3^), left postcentral gyrus (−16, −30, 76, cluster size = 368 mm^3^), and right insula (46, −18, 10, cluster size = 256 mm^3^). For each ROI, a correlation analysis was carried out between the mean signal change and the time series of every voxel of the whole brain. The resulting r values were converted using Fisher's *r*‐to‐*z* transformation to improve their Gaussian distribution.

### 2.3 Statistical analysis

The statistical analysis was carried out using SPSS 17.0. A two‐tailed independent samples *t* test was used for normally distributed variables, while the Mann–Whitney *U* test was used for non‐normally distributed data. Simultaneously, the chi‐squared test was used for categorical variables. A *p* value of <.05 was considered statistically significant. Voxel‐wise two‐sample *t* test embedded in DPABI was performed to evaluate the intergroup differences for each ROI after controlling for years of education. Significance was determined using the Gaussian random field (GRF) correction method with a *p* value of <.05 (voxel *p* < .001, cluster size > 29). The mean value of functional connections of functionally altered brain regions between groups was extracted from patients in the T2DM group. Partial correlation analyses were conducted between the mean values and clinical/cognitive variables after controlling for years of education.

## 3 RESULTS

### 3.1 Clinical and neuropsychological data

A total of 38 patients with T2DM and 33 HCs were enrolled in the final analysis. Demographic, clinical, and cognitive information from patients in the T2DM group and the HC group are listed in Table 1. There were no significant intergroup differences in age, sex, BMI, TC, TG, blood pressure, or cognitive score (*p* > .05). Education level was higher in patients in the HC group compared with patients in the T2DM group (*p* = .001). Compared with HC group, patients in the T2DM group had elevated levels of FBG and HbA1c (*p* < .001 for both).

### 3.2 Functional connectivity analysis

#### 3.2.1 ROI: right posterior cerebellum

**FIGURE 1:**
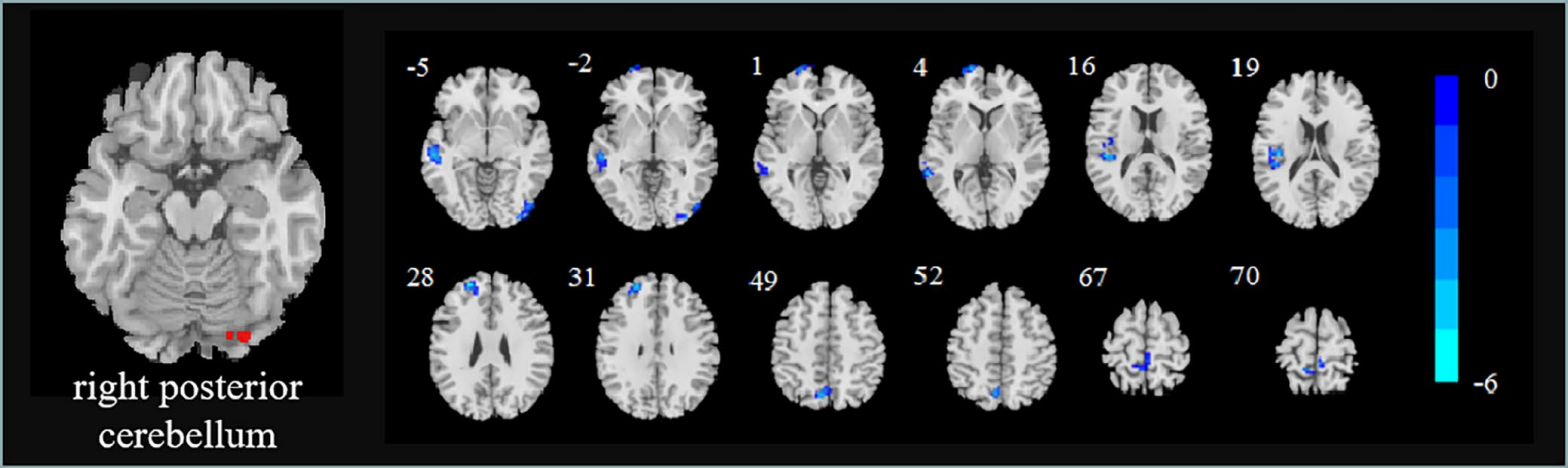
Significant differences were observed in right posterior cerebellum functional connectivity between the T2DM and HC groups. Thresholds were set using GRF correction at a *p* value of <.05 (voxel *p* < .001, cluster size > 29)

Compared with the HC group, the functional connections between the right posterior cerebellum and right middle/inferior occipital gyrus, left middle temporal gyrus, superior frontal gyrus, left middle frontal gyrus, left insula, left precuneus, and right paracentral lobule/left precuneus showed a significant decrease in the T2DM group (Figure 1 and Table 2).

**TABLE 2:** Aberrant functional connectivity in the T2DM group compared with the HC group

Seed ROI	Brain regions	Peak MNI coordinates			Voxels	BA	Peak t score
		X	Y	Z			
Right posterior cerebellum	R middle/inferior occipital gyrus	36	−93	−6	57	18/19	−4.55
	L middle temporal gyrus	−57	−24	−6	82	21/22	−4.99
	L superior frontal gyrus	−15	69	3	46	10	−4.84
	L insula	−39	−21	18	44	13	−5.04
	L middle frontal gyrus	−24	51	27	43	9/10	−5.34
	L precuneus	−6	−66	51	33	7	−4.94
	R paracentral lobule/L precuneus	−9	−42	69	33	4/5	−4.33
Right insula	L medial frontal gyrus	0	51	24	49	9	−4.20
	L supplementary motor area	−12	15	66	38	6	−5.98
Left lingual gyrus	R middle/inferior occipital gyrus	42	−87	3	71	18/19	−4.83

#### 3.2.2 ROI: right insula

**FIGURE 2:**
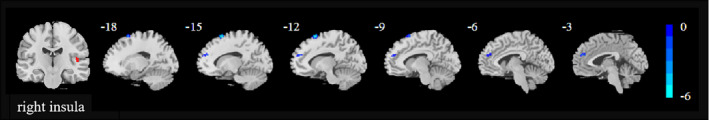
Significant differences were observed in right insula functional connectivity between the T2DM and HC groups. Thresholds were set using GRF correction at a corrected *p* value of <.05 (voxel *p* < .001, cluster size > 29)

The functional connections between the right insula and left medial frontal gyrus, supplementary motor area showed a significant decrease in the T2DM group (Figure 2 and Table 2).

#### 3.2.3 ROI: left lingual gyrus

**FIGURE 3:**
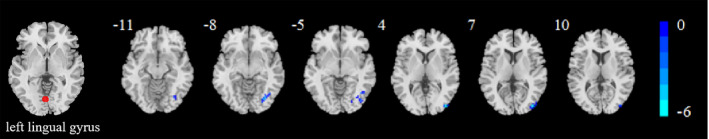
Significant differences were observed in left lingual gyrus functional connectivity between the T2DM and HC groups. Thresholds were set using GRF correction at a corrected *p* value of <.05 (voxel *p* < .001, cluster size > 29)

A significant decrease in the functional connection between the left lingual gyrus and right middle/inferior occipital gyrus was observed in the T2DM group (Figure 3 and Table 2).

#### 3.2.4 ROI: left postcentral gyrus

The functional connection between the left postcentral gyrus and other brain regions were not statistically significantly different between groups.

### 3.3 Correlation analysis

In the T2DM group, the functional connectivity between the right posterior cerebellum and left middle frontal gyrus, and between the right posterior cerebellum and left precuneus was negatively correlated with HbA1c (*r* = −.356, *p* = .03; *r* = −0.334, *p* = .043). However, the former significant correlation did not remain significant after Bonferroni correction. There was no correlation between abnormal functional connectivity and cognitive scores in patients with T2DM.

## 4 DISCUSSION

The present study used brain regions from the previous meta‐analysis as the ROIs to explore the functional connectivity patterns of these ROIs with the whole brain in patients with T2DM. We found multiple abnormal connections between different brain regions, especially the right cerebellar posterior lobe and extensive cerebral regions. In addition, the functional connectivity between the right posterior cerebellum and left middle frontal gyrus and left precuneus was negatively correlated with HbA1c in patients with T2DM. This suggests that the cerebellar–cerebral loop may be involved in neuropathological mechanisms of brain function in T2DM.

### 4.1 Decreased connectivity of the right posterior cerebellum in T2DM

Previous theories have suggested that the cerebellum is mainly associated with the somatosensory motor system. However, several recent studies have reported the location of motor‐related functional regions in the anterior cerebellum (Koziol et al., 2014; Stoodley & Schmahmann, 2018). In contrast, the posterior cerebellum is involved in several cognitive processes, including attention (Diano et al., 2016; Stoodley & Schmahmann, 2009), language processing (Guell et al., 2018; Stoodley & Schmahmann, 2009), working memory (Guell et al., 2018), and social cognition (Adamaszek et al., 2014; Guell et al., 2018). In contrast with the other three ROIs located in the cerebrum, the posterior cerebellum had the most widespread influence on the functioning of the whole brain in patients with T2DM in this study. Therefore, we speculated that the posterior cerebellum might participate in multiple pathways of brain dysfunction in T2DM, which deserves further verification in future research.

Studies of neuroanatomy have revealed a bidirectional projection between the cerebellum and the prefrontal cortex (Strick, Dum, & Fiez, 2009), suggesting a closed information loop of prefrontal–cerebellar in the human brain. This in turn provides the neural basis for the cerebellum to participate in multiple cognitive functions. Studies have confirmed that the prefrontal–cerebellar loop is related to working memory (Chanraud, Pitel, Muller‐Oehring, Pfefferbaum, & Sullivan, 2013) and the language network (Berl et al., 2014). Lower task‐based fMRI activation in the middle frontal gyrus is associated with poorer working memory, language, and executive function (Li et al., 2017). Our results show that the strength of functional connectivity between the right posterior cerebellum and the left middle frontal gyrus is negatively correlated with HbA1c, suggesting that the prefrontal–cerebellar loop may be involved in the neuropathology of T2DM.

The precuneus and middle temporal gyrus belong to default mode networks (DMN) and the posterior cerebellum is functionally coupled to DMN (Buckner et al., 2011). Multiple studies (Musen et al., 2012; Yang et al., 2016; Zhang et al., 2015) confirm abnormal functional connections within DMN and between DMN and other regions in patients with T2DM. In addition, one study (Yang et al., 2016) found that the connection between the bilateral posterior lobe of the cerebellum and DMN decreased and was negatively correlated with HbA1c in patients with T2DM. Our study found similar results and suggested that the decreased functional connection between the right posterior cerebellum and left precuneus was negatively correlated with HbA1c, which provides further evidence for the destruction of the connection between the cerebellum and DMN in patients with T2DM, especially the disconnection between the right posterior cerebellum and left precuneus. Patients with depression have abnormal functional connections between the cerebellum and precuneus and may predict suicidal tendencies (Zhang et al., 2016). Several studies (Stuart & Baune, 2012; O'Connor et al., 2009) suggest that the relationship between depression and T2DM is bidirectional or comorbidity. Therefore, we speculate that disconnection between the right posterior cerebellum and left precuneus may be related to emotional abnormalities in patients with T2DM.

### 4.2 Decreased connectivity of the right insula in T2DM

The eating patterns in T2DM are commonly altered and sometimes unhealthy, and the insula is closely related to food intake. It has been found that the insula, which contains the primary taste cortex, recognizes aromas, and has the ability to integrate smell (Cornier, 2011; Hinton et al., 2004). In addition, increased neural activity in the insula after stimulation by food cues involves the process of food compensation and manages feeding behavior (Cornier et al., 2012). The medial frontal gyrus (BA9) belongs to the dorsolateral prefrontal lobe. Current studies have demonstrated that (Gluck, Viswanath, & Stinson, 2017) the dorsolateral prefrontal lobes played an important role in diet control and food craving. Thus, the reduced functional connection between the insula and the medial frontal gyrus may be associated with a decreased ability to control food intake and abnormal eating behaviors in patients with T2DM.

A meta‐analysis of 1,768 functional neuroimaging experiments confirmed the involvement of the insula in motor function (Kurth, Zilles, Fox, Laird, & Eickhoff, 2010) and a direct structural connection between the insula and the motor cortices (Showers & Lauer, 1961). Our results show that decreased functional connectivity between the insula and the supplementary motor area (BA6) might impair motor function in patients with T2DM. It has been reported that patients with T2DM have poorer muscle performance (Bassil et al., 2011) and leg muscle strength (Park et al., 2006). In addition, one study found that female patients with T2DM have decreased fine motor function (Espeland et al., 2011). This research is consistent with our speculation.

### 4.3 Decreased connectivity of the left lingual gyrus in T2DM

Multiple studies (Cui et al., 2014; Wang et al., 2017) have confirmed the visual processing area of the occipital lobe is the most vulnerable region of the brain to T2DM. Cui et al. (2016) found diffusely decreased connectivity in the lingual gyrus‐related visual network in patients with T2DM. Our results were consistent with their findings. The lingual gyrus and middle occipital gyrus are important nodes of the visual network, which are crucial for the visual information processing relating to visual cognition (Zhen et al., 2018). Reduced functional connection between the lingual gyrus and middle/inferior occipital gyrus indicates visual impairment in patients with T2DM. However, due to the presence of diabetic retinopathy patients in this study, we cannot determine whether this abnormal functional connection occurs before or reflects the neural basis of retinopathy. In future studies, we will try to clarify the neural mechanism of this abnormality.

There are some limitations of the present study. First, the patient cohort in this study was relatively small. Increasing the sample size may increase the credibility of our results. Second, the blood glucose was not measured directly before neuropsychological assessment and MRI examination, further studies should take it into consideration to better elucidate the relationship between glycemic control and neural dysfunction. Third, most patients with T2DM in this study were revisiting and had a long disease duration, so our results may not be extended to patients with a short disease duration. Fourth, because all the subjects included in the previous meta‐analysis were Chinese, the results of this study may not be reproducible in other countries or ethnic populations. Finally, we did not control the T2DM complications and treatment methods during the enrollment process, which may have had a certain bias on the results. For example, metformin may affect the cognitive function of patients with diabetes (Moore et al., 2013).

## 5 CONCLUSIONS

In this study, data‐driven ROI was used to identify extensive changes in functional connectivity in brain regions of patients with T2DM, especially in the cerebellar–cerebral circuit. It is suggested that the cerebellar–cerebral circuit may be involved in the neuropathological basis of brain dysfunction in patients with T2DM, which provides new insight into the neural mechanisms of brain dysfunction in patients with T2DM from the perspective of the cerebellum.

## CONFLICT OF INTEREST

The authors declare no potential conflicts of interest with respect to the authorship and/or publication of this article.

## AUTHOR CONTRIBUTIONS

Dongsheng Zhang drafted the manuscript, designed the experiment, and performed the statistical analysis. Jie Gao contributed to performing the experiments and revised the manuscript. Min Tang, Xia Zhe, and Xuejiao Yan collected the data. Miao Cheng and Weibo Chen provided technical support. Xiaoling Zhang made contributions to the design of the experiment and revised the manuscript. All authors read and approved the final manuscript.

## Data Availability

The data that support the findings of this study are available on requests from the corresponding author.

## References

[brb31725-bib-0001] Adamaszek, M. , D'Agata, F. , Kirkby, K. C. , Trenner, M. U. , Sehm, B. , Steele, C. J. , … Strecker, K. (2014). Impairment of emotional facial expression and prosody discrimination due to ischemic cerebellar lesions. Cerebellum, 13, 338–345. 10.1007/s12311-013-0537-0 24281851

[brb31725-bib-0002] Avissar, M. , Powell, F. , Ilieva, I. , Respino, M. , Gunning, F. M. , Liston, C. , & Dubin, M. J. (2017). Functional connectivity of the left DLPFC to striatum predicts treatment response of depression to TMS. Brain Stimulation, 10, 919–925. 10.1016/j.brs.2017.07.002 28747260 PMC5568496

[brb31725-bib-0003] Bassil, M. , Marliss, E. B. , Morais, J. A. , Pereira, S. , Chevalier, S. , & Gougeon, R. (2011). Postprandial hyperaminoacidaemia overcomes insulin resistance of protein anabolism in men with type 2 diabetes. Diabetologia, 54, 648–656. 10.1007/s00125-010-1980-9 21109998

[brb31725-bib-0004] Berl, M. M. , Mayo, J. , Parks, E. N. , Rosenberger, L. R. , VanMeter, J. , Ratner, N. B. , … Gaillard, W. D. (2014). Regional differences in the developmental trajectory of lateralization of the language network. Human Brain Mapping, 35, 270–284. 10.1002/hbm.22179 23033058 PMC3578038

[brb31725-bib-0005] Biessels, G. J. , Deary, I. J. , & Ryan, C. M. (2008). Cognition and diabetes: A lifespan perspective. The Lancet Neurology, 7, 184–190. 10.1016/S1474-4422(08)70021-8 18207116

[brb31725-bib-0006] Biswal, B. , Yetkin, F. Z. , Haughton, V. M. , & Hyde, J. S. (1995). Functional connectivity in the motor cortex of resting human brain using echo‐planar MRI. Magnetic Resonance in Medicine, 34, 537–541. 10.1002/mrm.1910340409 8524021

[brb31725-bib-0007] Buckner, R. L. , Krienen, F. M. , Castellanos, A. , Diaz, J. C. , & Yeo, B. T. (2011). The organization of the human cerebellum estimated by intrinsic functional connectivity. Journal of Neurophysiology, 106, 2322–2345. 10.1152/jn.00339.2011 21795627 PMC3214121

[brb31725-bib-0008] Chanraud, S. , Pitel, A. L. , Muller‐Oehring, E. M. , Pfefferbaum, A. , & Sullivan, E. V. (2013). Remapping the brain to compensate for impairment in recovering alcoholics. Cerebral Cortex, 23, 97–104. 10.1093/cercor/bhr381 22275479 PMC3513953

[brb31725-bib-0009] Cheke, L. G. , Bonnici, H. M. , Clayton, N. S. , & Simons, J. S. (2017). Obesity and insulin resistance are associated with reduced activity in core memory regions of the brain. Neuropsychologia, 96, 137–149. 10.1016/j.neuropsychologia.2017.01.013 28093279 PMC5317178

[brb31725-bib-0010] Chen, Y. C. , Xia, W. , Qian, C. , Ding, J. , Ju, S. , & Teng, G. J. (2015). Thalamic resting‐state functional connectivity: Disruption in patients with type 2 diabetes. Metabolic Brain Disease, 30, 1227–1236. 10.3389/fnagi.2015.00233 26116166

[brb31725-bib-0011] Chen, Z. , Liu, M. , & Ma, L. (2014). Resting‐state brain functional magnetic resonance imaging in patients with type 2 diabetes mellitus. Nan Fang Yi Ke Da Xue Xue Bao, 34, 1083–1091. 10.3969/j.issn.1673-4254.2014.08.02 25176072

[brb31725-bib-0012] Cole, D. M. , Smith, S. M. , & Beckmann, C. F. (2010). Advances and pitfalls in the analysis and interpretation of resting‐state FMRI data. Frontiers in Systems Neuroscience, 4, 8 10.3389/fnsys.2010.00008 20407579 PMC2854531

[brb31725-bib-0013] Cornier, M. A. (2011). Is your brain to blame for weight regain? Physiology & Behavior, 104, 608–612. 10.1016/j.physbeh.2011.04.003 21496461 PMC3139793

[brb31725-bib-0014] Cornier, M. A. , Melanson, E. L. , Salzberg, A. K. , Bechtell, J. L. , & Tregellas, J. R. (2012). The effects of exercise on the neuronal response to food cues. Physiology & Behavior, 105, 1028–1034. 10.1016/j.physbeh.2011.11.023 22155218 PMC3260391

[brb31725-bib-0015] Cui, Y. , Jiao, Y. , Chen, H. J. , Ding, J. , Luo, B. , Peng, C. Y. , … Teng, G. J. (2015). Aberrant functional connectivity of default‐mode network in type 2 diabetes patients. European Radiology, 25(11), 3238–3246. 10.1007/s00330-015-3746-8 25903712 PMC4595523

[brb31725-bib-0016] Cui, Y. , Jiao, Y. , Chen, Y. C. , Wang, K. , Gao, B. , Wen, S. , … Teng, G. J. (2014). Altered spontaneous brain activity in type 2 diabetes: A resting‐state functional MRI study. Diabetes, 63, 749–760. 10.2337/db13-0519 24353185

[brb31725-bib-0017] Cui, Y. , Li, S. F. , Gu, H. , Hu, Y. Z. , Liang, X. , Lu, C. Q. , … Teng, G. J. (2016). Disrupted brain connectivity patterns in patients with type 2 diabetes. American Journal of Neuroradiology, 37, 2115–2122. 10.3174/ajnr.A4858 27365332 PMC5201447

[brb31725-bib-0018] De Felice, F. G. , & Lourenco, M. V. (2015). Brain metabolic stress and neuroinflammation at the basis of cognitive impairment in Alzheimer's disease. Frontiers in Aging Neuroscience, 7, 94 10.3389/fnagi.2015.00094 26042036 PMC4436878

[brb31725-bib-0019] Diano, M. , D'Agata, F. , Cauda, F. , Costa, T. , Geda, E. , Sacco, K. , … Geminiani, G. C. (2016). Cerebellar clustering and functional connectivity during pain processing. Cerebellum, 15, 343–356. 10.1007/s12311-015-0706-4 26202672

[brb31725-bib-0020] Du, Y. , Fryer, S. L. , Fu, Z. , Lin, D. , Sui, J. , Chen, J. , … Calhoun, V. D. (2018). Dynamic functional connectivity impairments in early schizophrenia and clinical high‐risk for psychosis. NeuroImage, 180, 632–645. 10.1016/j.neuroimage.2017.10.022 29038030 PMC5899692

[brb31725-bib-0021] Du, Y. , Fu, Z. , & Calhoun, V. D. (2018). Classification and prediction of brain disorders using functional connectivity: Promising but challenging. Frontiers in Neuroscience, 12, 525 10.3389/fnins.2018.00525. 30127711 PMC6088208

[brb31725-bib-0022] Espeland, M. A. , Miller, M. E. , Goveas, J. S. , Hogan, P. E. , Coker, L. H. , Williamson, J. , … Resnick, S. M. (2011). Cognitive function and fine motor speed in older women with diabetes mellitus: Results from the women's health initiative study of cognitive aging. Journal of Women's Health, 20, 1435–1443. 10.1089/jwh.2011.2812 PMC3186442 21819251

[brb31725-bib-0023] Gluck, M. E. , Viswanath, P. , & Stinson, E. J. (2017). Obesity, appetite, and the prefrontal cortex. Current Obesity Reports, 6, 380–388. 10.1007/s13679-017-0289-0 29071480

[brb31725-bib-0024] Guell, X. , Gabrieli, J. D. E. , & Schmahmann, J. D. (2018). Triple representation of language, working memory, social and emotion processing in the cerebellum: Convergent evidence from task and seed‐based resting‐state fMRI analyses in a single large cohort. NeuroImage, 172, 437–449. 10.1016/j.neuroimage.2018.01.082 29408539 PMC5910233

[brb31725-bib-0025] Hinton, E. C. , Parkinson, J. A. , Holland, A. J. , Arana, F. S. , Roberts, A. C. , & Owen, A. M. (2004). Neural contributions to the motivational control of appetite in humans. The European Journal of Neuroscience, 20, 1411–1418. 10.1111/j.1460-9568.2004.03589.x 15341613

[brb31725-bib-0026] Iyalomhe, O. , Swierczek, S. , Enwerem, N. , Chen, Y. , Adedeji, M. O. , Allard, J. , … Obisesan, T. O. (2017). The role of hypoxia‐inducible factor 1 in mild cognitive impairment. Cellular and Molecular Neurobiology, 37, 969–977. 10.1007/s10571-016-0440-6 27858285 PMC5435557

[brb31725-bib-0027] Koziol, L. F. , Budding, D. , Andreasen, N. , D'Arrigo, S. , Bulgheroni, S. , Imamizu, H. , … Yamazaki, T. (2014). Consensus paper: The cerebellum's role in movement and cognition. Cerebellum, 13, 151–177. 10.1007/s12311-013-0511-x 23996631 PMC4089997

[brb31725-bib-0028] Kurth, F. , Zilles, K. , Fox, P. T. , Laird, A. R. , & Eickhoff, S. B. (2010). A link between the systems: Functional differentiation and integration within the human insula revealed by meta‐analysis. Brain Structure & Function, 214, 519–534. 10.1007/s00429-010-0255-z 20512376 PMC4801482

[brb31725-bib-0029] Li, X. , Wang, W. , Wang, A. , Li, P. , Zhang, J. , Tao, W. , & Zhang, Z. (2017). Vulnerability of the frontal and parietal regions in hypertensive patients during working memory task. Journal of Hypertension, 35, 1044–1051. 10.1097/HJH.0000000000001250 28118278

[brb31725-bib-0030] Liu, D. , Duan, S. , Zhang, J. , Zhou, C. , Liang, M. , Yin, X. , … Wang, J. (2016). Aberrant brain regional homogeneity and functional connectivity in middle‐aged T2DM patients: A resting‐state functional MRI study. Frontiers in Human Neuroscience, 10, 490 10.3389/fnhum.2016.00490 27729856 PMC5037166

[brb31725-bib-0031] Macpherson, H. , Formica, M. , Harris, E. , & Daly, R. M. (2017). Brain functional alterations in type 2 diabetes ‐ a systematic review of fMRI studies. Frontiers in Neuroendocrinology, 47, 34–46. 10.1016/j.yfrne.2017.07.001 28687473

[brb31725-bib-0032] Moore, E. M. , Mander, A. G. , Ames, D. , Kotowicz, M. A. , Carne, R. P. , Brodaty, H. , … Investigators, A. (2013). Increased risk of cognitive impairment in patients with diabetes is associated with metformin. Diabetes Care, 36, 2981–2987. 10.2337/dc13-0229 24009301 PMC3781568

[brb31725-bib-0033] Musen, G. , Jacobson, A. M. , Bolo, N. R. , Simonson, D. C. , Shenton, M. E. , McCartney, R. L. , … Hoogenboom, W. S. (2012). Resting‐state brain functional connectivity is altered in type 2 diabetes. Diabetes, 61, 2375–2379. 10.2337/db11-1669 22664957 PMC3425418

[brb31725-bib-0034] O'Connor, P. J. , Crain, A. L. , Rush, W. A. , Hanson, A. M. , Fischer, L. R. , & Kluznik, J. C. (2009). Does diabetes double the risk of depression? The Annals of Family Medicine, 7, 328–335. 10.1370/afm.964 19597170 PMC2713167

[brb31725-bib-0035] Park, S. W. , Goodpaster, B. H. , Strotmeyer, E. S. , de Rekeneire, N. , Harris, T. B. , Schwartz, A. V. , … Newman, A. B. (2006). Decreased muscle strength and quality in older adults with type 2 diabetes: The health, aging, and body composition study. Diabetes, 55, 1813–1818. 10.2337/db05-1183 16731847

[brb31725-bib-0036] Peng, J. , Qu, H. , Peng, J. , Luo, T.‐Y. , Lv, F.‐J. , chen, L. I. , … Cheng, Q.‐F. (2016). Abnormal spontaneous brain activity in type 2 diabetes with and without microangiopathy revealed by regional homogeneity. European Journal of Radiology, 85, 607–615. 10.1016/j.ejrad.2015.12.024 26860674

[brb31725-bib-0037] Showers, M. J. , & Lauer, E. W. (1961). Somatovisceral motor patterns in the insula. The Journal of Comparative Neurology, 117, 107–115. 10.1002/cne.901170109 13912292

[brb31725-bib-0038] Stoodley, C. J. , & Schmahmann, J. D. (2009). Functional topography in the human cerebellum: A meta‐analysis of neuroimaging studies. NeuroImage, 44, 489–501. 10.1016/j.neuroimage.2008.08.039 18835452

[brb31725-bib-0039] Stoodley, C. J. , & Schmahmann, J. D. (2018). Functional topography of the human cerebellum. Handbook of Clinical Neurology, 154, 59–70. 10.1016/B978-0-444-63956-1.00004-7 29903452

[brb31725-bib-0040] Strick, P. L. , Dum, R. P. , & Fiez, J. A. (2009). Cerebellum and nonmotor function. Annual Review of Neuroscience, 32, 413–434. 10.1146/annurev.neuro.31.060407.125606 19555291

[brb31725-bib-0041] Stuart, M. J. , & Baune, B. T. (2012). Depression and type 2 diabetes: Inflammatory mechanisms of a psychoneuroendocrine co‐morbidity. Neuroscience & Biobehavioral Reviews, 36, 658–676. 10.1016/j.neubiorev.2011.10.001 22020230

[brb31725-bib-0042] Sun, Q. , Chen, G. Q. , Wang, X. B. , Yu, Y. , Hu, Y. C. , Yan, L. F. , … Cui, G. B. (2018). Alterations of white matter integrity and hippocampal functional connectivity in type 2 diabetes without mild cognitive impairment. Frontiers in Neuroanatomy, 12, 21 10.3389/fnana.2018.00021 29615873 PMC5869188

[brb31725-bib-0043] Wang, C. X. , Fu, K. L. , Liu, H. J. , Xing, F. , & Zhang, S. Y. (2014). Spontaneous brain activity in type 2 diabetics revealed by amplitude of low‐frequency fluctuations and its association with diabetic vascular disease: A resting‐state fMRI study. PLoS One, 9, e108883 10.1371/journal.pone.0108883 25272033 PMC4182760

[brb31725-bib-0044] Wang, Z. L. , Zou, L. , Lu, Z. W. , Xie, X. Q. , Jia, Z. Z. , Pan, C. J. , … Ge, X. M. (2017). Abnormal spontaneous brain activity in type 2 diabetic retinopathy revealed by amplitude of low‐frequency fluctuations: A resting‐state fMRI study. Clinical Radiology, 72(4), 340.e1–340.e7. 10.1016/j.crad.2016.11.012 28041652

[brb31725-bib-0045] Woolley, J. D. , Gorno‐Tempini, M. L. , Seeley, W. W. , Rankin, K. , Lee, S. S. , Matthews, B. R. , & Miller, B. L. (2007). Binge eating is associated with right orbitofrontal‐insular‐striatal atrophy in frontotemporal dementia. Neurology, 69, 1424–1433. 10.1212/01.wnl.0000277461.06713.23 17909155

[brb31725-bib-0046] Xia, W. , Luo, Y. , Chen, Y. C. , Zhang, D. , Bo, F. , Zhou, P. , … Ma, J. (2018). Disrupted functional connectivity of the amygdala is associated with depressive mood in type 2 diabetes patients. Journal of Affective Disorders, 228, 207–215. 10.1016/j.jad.2017.12.012 29272791

[brb31725-bib-0047] Xia, W. , Wang, S. , Sun, Z. , Bai, F. , Zhou, Y. I. , Yang, Y. , … Yuan, Y. (2013). Altered baseline brain activity in type 2 diabetes: A resting‐state fMRI study. Diabetologia, 56, S41–S42. 10.1016/j.psyneuen.2013.05.012 23786881

[brb31725-bib-0048] Yang, S. Q. , Xu, Z. P. , Xiong, Y. , Zhan, Y. F. , Guo, L. Y. , Zhang, S. , … Zhu, W. Z. (2016). Altered intranetwork and internetwork functional connectivity in type 2 diabetes mellitus with and without cognitive impairment. Scientific Reports, 6, 32980 10.1038/srep32980 27622870 PMC5020685

[brb31725-bib-0049] Zhang, D. S. , Gao, J. , Zhe, X. , Yan, X. J. , Tang, M. , Yang, J. , & Zhang, X. L. (2018). Altered spontaneous brain activity in type 2 diabetes mellitus: An activation likelihood estimation Meta‐analysis (article in Chinese). Chinese Journal of Radiology, 52, 241–246. 10.3760/cma.j.issn.1005-1201.2018.04.001

[brb31725-bib-0050] Zhang, H. , Hao, Y. , Manor, B. , Novak, P. , Milberg, W. , Zhang, J. , … Novak, V. (2015). Intranasal insulin enhanced resting‐state functional connectivity of hippocampal regions in type 2 diabetes. Diabetes, 64, 1025–1034. 10.2337/db14-1000 25249577 PMC4338591

[brb31725-bib-0051] Zhang, S. , Chen, J. M. , Kuang, L. , Cao, J. , Zhang, H. , Ai, M. , … Fang, W. D. (2016). Association between abnormal default mode network activity and suicidality in depressed adolescents. BMC Psychiatry, 16, 337 10.1186/s12888-016-1047-7 27688124 PMC5041526

[brb31725-bib-0052] Zhen, D. , Xia, W. , Yi, Z. Q. , Zhao, P. W. , Zhong, J. G. , Shi, H. C. , … Pan, P. L. (2018). Alterations of brain local functional connectivity in amnestic mild cognitive impairment. Translational Neurodegeneration, 7, 26 10.1186/s40035-018-0134-8 30443345 PMC6220503

[brb31725-bib-0053] Zhou, X. , Zhang, J. , Chen, Y. , Ma, T. , Wang, Y. , Wang, J. , & Zhang, Z. (2014). Aggravated cognitive and brain functional impairment in mild cognitive impairment patients with type 2 diabetes: A resting‐state functional MRI study. Journal of Alzheimer's Disease, 41, 925–935. 10.3233/JAD-132354 24705547

